# Phytochemistry, Bioactivity, and Toxicological Duality of *Oxytropis glabra* DC: A Review

**DOI:** 10.3390/molecules31010044

**Published:** 2025-12-22

**Authors:** Karlygash Raganina, Akerke Amirkhanova, Sholpan Akhelova, Aiman Berdgaleyeva, Meruyert Amantayeva, Elmira Kartbayeva, Aigul Kaldybayeva, Madi Nurlybayev, Yerbol Ikhsanov, Nurzhan Iztileu, Zhanserik Shynykul, Moldir Koilybayeva

**Affiliations:** 1 School of Pharmacy, S.D. Asfendiyarov Kazakh National Medical University, Tole-bi 94, Almaty 050012, Kazakhstan; raganina.k@kaznmu.kz (K.R.); amirhanova.a@kaznmu.kz (A.A.); amantaeva.meruert@kaznmu.kz (M.A.);; 2 Department of Pharmaceutical Disciplines, Astana Medical University, Astana 010000, Kazakhstan; sholpanakhelova@gmail.com (S.A.); iztileu.n@amu.kz (N.I.); 3 Department of Pharmaceutical Disciplines, Marat Ospanov West Kazakhstan Medical University, Maresyev 68, Aktobe 030019, Kazakhstan; bak5393@mail.ru; 4 Higher School of Medicine, Al-Farabi Kazakh National University, Tole-bi 96, Almaty 050040, Kazakhstan; madi.nurlybayev@gmail.com; 5 Department of Pharmaceutical and Toxicological Chemistry, S.D. Asfendiyarov Kazakh National Medical University, Tole-bi 94, Almaty 050012, Kazakhstan; 6 Department of Chemistry and Technology of Organic Substances, Natural Compounds and Polymers, Faculty of Chemistry and Chemical Technology, Al-Farabi Kazakh National University, Almaty 050040, Kazakhstan; erbol.ih@gmail.com

**Keywords:** *Oxytropis glabra*, alkaloids, swainsonine, flavonoids, toxicity, bioactive metabolites, pharmacological activity, phytochemistry

## Abstract

*Oxytropis glabra* DC, a Fabaceae species distributed across Central Asia, is characterized by a dual biological profile encompassing pronounced toxicity alongside promising pharmacological potential. This review synthesizes current knowledge on its phytochemistry, bioactivity, and toxicological liabilities to clarify the plant’s risk–benefit landscape. The objectives are to summarize the dominant classes of metabolites identified in *O. glabra*, evaluate their toxicological and therapeutic relevance, and identify key gaps limiting translational research. *O. glabra* contains a diverse array of secondary metabolites, with quinolizidine and indolizidine alkaloids, including swainsonine, anagyrine, thermopsine, and sparteine, representing the primary determinants of toxicity. These compounds are associated with teratogenicity, neurotoxicity, and locoism through mechanisms involving α-mannosidase inhibition, disruption of glycoprotein processing, and impaired lysosomal homeostasis. In contrast, flavonoids such as quercetin, isoquercitrin, and kaempferol derivatives exhibit antioxidant, anti-inflammatory, hepatoprotective, and cardioprotective effects, while triterpenoid saponins and fatty acids contribute additional cytoprotective and metabolic activities. Despite extensive reports on both toxic and bioactive constituents, critical gaps remain regarding chemotype variability, dose–response relationships, and pharmacokinetics, which currently constrain therapeutic exploitation. Future research should prioritize defining safe exposure thresholds, elucidating structure–activity relationships, and developing standardized extracts or optimized derivatives that balance efficacy and safety. This integrative perspective highlights *O. glabra* as a chemically rich but biologically ambivalent species whose toxicological risks and pharmacological opportunities warrant systematic mechanistic investigation.

## 1. Introduction

The exploration of phytochemicals derived from various plant taxa within the realm of ethnomedicine represents an increasingly prominent domain that integrates ancestral knowledge with contemporary pharmacological inquiry [1]. Ethnomedicine encapsulates millennia of empirical engagement with medicinal flora, thereby providing significant insights regarding species that may exhibit therapeutic efficacy [2]. Although certain botanical specimens are classified as toxic to both animals and humans, their examination remains critical, as the phytochemical constituents responsible for their toxicity may also manifest considerable pharmacological activity when administered at regulated dosages [3]. For example, numerous well-established pharmaceuticals have been synthesized from toxic flora, including cardiac glycosides obtained from *Digitalis purpurea* and atropine derived from *Atropa belladonna* [4,5]. Similarly, *Oxytropis glabra* DC, which precipitates neurological afflictions in herbivorous animals, harbors alkaloids and additional bioactive metabolites that may act as precursors for the development of novel therapeutic modalities. The investigation of these phytoconstituents enables scholars to isolate, characterize, and evaluate compounds with prospective anticancer, antimicrobial, or neuroprotective attributes [6]. Furthermore, such research endeavors facilitate the conservation of ethnobotanical wisdom and biodiversity while augmenting comprehension of the intricate relationships between toxic and beneficial effects. Consequently, the scrutiny of phytochemicals from both medicinal and toxic plant species is of paramount importance for the identification of novel bioactive compounds, the assurance of safe applications, and the advancement of drug discovery and pharmacological innovation.

The genus *Oxytropis* (Fabaceae) encompasses over 300 species that are distributed throughout the temperate zones of Asia, Europe, and North America, with a particularly pronounced diversity noted in Central Asia [6]. Species within this genus are recognized for their remarkable resilience to the extreme conditions prevalent in alpine and steppe ecosystems, and they have been historically utilized in traditional medicine due to their anti-inflammatory, antimicrobial, antioxidant, and hepatoprotective properties [7,8]. Phytochemical investigations of *Oxytropis* species have unveiled a complex array of flavonoids, alkaloids, saponins, and polysaccharides, many of which exhibit significant biological activities pertinent to contemporary pharmacological applications [9]. Among the extensively researched compounds is swainsonine (SW), an indolizidine alkaloid implicated in the neurotoxic phenomenon of “locoism” in livestock, yet simultaneously acknowledged for its potential antitumor and immunomodulatory effects when administered at regulated doses [10]. Additional species within the genus, including *O. falcata*, *O. racemosa*, and *O. ochrocephala*, have shown encouraging outcomes in experimental models addressing inflammation, oxidative stress, and infectious processes [6,11,12,13]. Within this heterogeneous genus, *O. glabra* holds a unique position as a prevalent species in Central Asia, particularly in the steppes of Kazakhstan and Mongolia, where it is both ecologically important and pharmacologically noteworthy [14,15]. Although it is recognized for inducing chronic neurological disorders in grazing animals due to the accumulation of SW, preliminary investigations indicate that *O. glabra* also possesses phenolic and flavonoid components that may confer therapeutic benefits [6,14]. Consequently, comprehensive phytochemical and pharmacological investigations of *O. glabra* are imperative to elucidate its dual toxicological and medicinal capabilities and to assess its potential as a source of novel bioactive compounds.

The study of phytoconstituents in *O. glabra* is of particular importance because, despite its recognized toxicity to grazing animals, this species remains largely unexplored from a phytochemical and pharmacological perspective. Preliminary reports indicate that members of the *Oxytropis* genus contain diverse classes of bioactive compounds, including alkaloids, flavonoids, saponins, and phenolic acids, which are known to contribute to a broad spectrum of biological effects such as antioxidant, antimicrobial, and anti-inflammatory activities [16,17,18,19,20,21,22,23,24]. However, specific data on the chemical composition and pharmacological profile of *O. glabra* are still scarce, highlighting the need for systematic investigation. Understanding its phytochemical makeup may reveal compounds with therapeutic potential, as some toxic constituents, like SW, have demonstrated valuable pharmacological properties when properly isolated and studied. The purpose of this review is therefore to summarize the existing information on the *Oxytropis* genus with particular attention to *O. glabra*, focusing on its botanical characteristics, geographical distribution, traditional and ethnomedicinal relevance, known chemical constituents, and reported biological activities. This review also aims to identify current research gaps and emphasize the necessity for future studies to explore the dual toxicological and pharmacological potential of *O. glabra*, which could ultimately contribute to the discovery of new bioactive molecules and the advancement of natural product-based drug development.

## 2. Methods

A comprehensive search of scientific databases, including Google Scholar and PubMed, was conducted to collect published information related to *O. glabra*. The search strategy employed combinations of keywords such as “*Oxytropis glabra*”, “*Oxytropis glabra* phytochemicals”, “*Oxytropis glabra* compounds”, “*Oxytropis glabra* pharmacological”, “*Oxytropis glabra* toxicity”, and “*Oxytropis glabra* traditional uses”. The total number of search results retrieved from each database is summarized in Table 1. The inclusion criteria encompassed studies focusing on the botanical description, geographical distribution, ethnomedicinal relevance, chemical constituents, and biological or toxicological activities of *O. glabra*. Additional references were obtained by manually screening the bibliographies of the selected articles.

**Figure 1:**
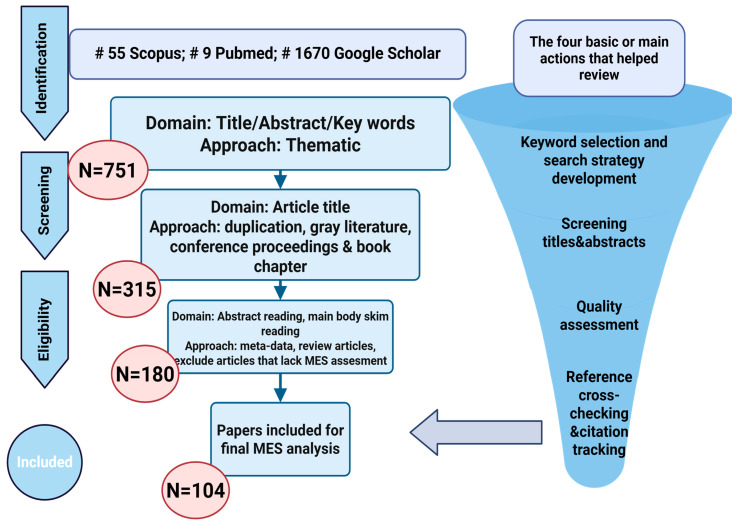
PRISMA 2020 flow diagram illustrating the literature search strategy, screening, eligibility assessment, and study selection process for the systematic review of *O. glabra* [25].

This systematic review was conducted in accordance with the PRISMA 2020 guidelines [25]. The identification, screening, eligibility assessment, and final inclusion steps followed the standardized PRISMA process. The PRISMA 2021 flow diagram, illustrating the progression from the initial search to the final selection of studies, is presented in Figure 1.

The literature search initially identified 1734 records (55 from Scopus, 9 from PubMed, and 1670 from Google Scholar). After the first screening of titles, abstracts, and keywords using a thematic approach, 751 records were retained. Subsequent screening of article titles, including removal of duplicates, gray literature, conference abstracts, and book chapters, further reduced the dataset to 315 articles. During the eligibility phase, 180 full-text articles were examined through abstract reading and targeted body-text screening, with exclusions applied to meta-analyses, review papers, and articles lacking relevance to MES (morphological, ethnomedicinal, or scientific) assessment.

Following this multistage evaluation, 104 studies met all eligibility criteria and were included in the final synthesis. These publications encompass ethnobotanical, phytochemical, pharmacological, and toxicological reports relevant to Oxytropis species, with particular emphasis on O. glabra, and are listed in References [1,2,3,4,5,6,7,8,9,10,11,12,13,14,15,16,17,18,19,20,21,22,23,24,25,26,27,28,29,30,31,32,33,34,35,36,37,38,39,40,41,42,43,44,45,46,47,48,49,50,51,52,53,54,55,56,57,58,59,60,61,62,63,64,65,66,67,68,69,70,71,72,73,74,75,76,77,78,79,80,81,82,83,84,85,86,87,88,89,90,91,92,93,94,95,96,97,98,99,100,101].

## 3. Distribution and Botanical Characterization

**Figure 2:**
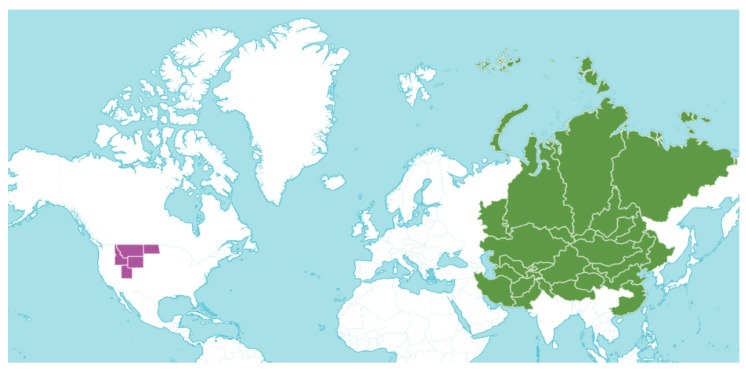
Global geographical distribution of *O. glabra*. Regions highlighted in green indicate areas where *O. glabra* has been reported to occur naturally or has been documented in the literature, primarily across Central Asia, Siberia, and adjacent regions. Non-shaded areas represent regions with no confirmed records of *O. glabra* distribution [26].

The taxonomic group *Oxytropis* DC. is predominantly distributed across the temperate and subarctic regions of Eurasia, with the highest species diversity occurring in Siberia, Mongolia, Kazakhstan, and northern China, extending westward into Central Asia and eastward toward the Russian Far East [6,14]. Species classified under the genus *Oxytropis* exhibit remarkable adaptations to extreme habitats, including alpine meadows, arid steppes, and rugged mountainous landscapes, where they are instrumental in maintaining soil integrity and ecological equilibrium. Among these species, *O. glabra* ranks as one of the most prevalent within the geographic confines of Kazakhstan and Mongolia (Figure 2) [26]. This species is typically associated with arid steppes, foothill regions, and semi-desert environments, flourishing in various soil types, including sandy, loamy, and stony substrates. *O. glabra* plays a crucial role in enhancing regional biodiversity and functions as a significant bioindicator of ecological resilience in harsh continental climatic conditions [27,28].

*O. glabra* is a perennial herbaceous organism characterized by a profound root system and a lignified rhizome that facilitates its persistence in arid ecosystems. The aerial stems are numerous, either ascending or erect, with a potential height ranging from 15 to 40 cm. These stems are densely adorned with fine, appressed, silvery trichomes (sericeous), imparting a distinctive grayish hue to the plant. The leaves exhibit an imparipinnate configuration, generally measuring 5–10 cm in length, consisting of 9–17 narrowly oblong or linear leaflets, each approximately 10–25 mm in length and 2–5 mm in width. The leaflets are enveloped in silky trichomes on both surfaces, while the petiole is short and robust. The stipules are lanceolate, pubescent, and partially fused with the petiole. These morphological adaptations enable the organism to mitigate water loss and endure the extreme solar radiation and wind exposure characteristic of steppe environments [27,28].

The inflorescence of *O. glabra* manifests as a dense raceme, typically comprising 10–25 papilionaceous flowers exhibiting blue to violet pigmentation. The calyx is tubular, measuring 8–10 mm in length, covered with appressed white or gray trichomes, and divided into linear–lanceolate lobes. The corolla generally reaches a length of 15–18 mm, with a standard petal (vexillum) that is marginally longer than the wings and keel. The stamens exhibit a diadelphous arrangement (9 + 1), forming a tubular structure encasing the ovary. The ovary is sessile and densely hairy, housing numerous ovules, while the style is slender and exhibits a slight curvature. The fruit is a linear–ovoid legume, measuring 10–15 mm in length, densely hairy, and dehiscent upon maturation. The seeds are diminutive, brownish-yellow, and exhibit a renal shape [17,18,19]. Morphologically, *O. glabra* displays characteristics emblematic of xerophytic legumes, including dense pubescence and compact growth, which enhance its survival in nutrient-deficient and arid habitats. These botanical attributes, in conjunction with its distinctive phytochemical composition, render *O. glabra* a species of significant interest for ecological, toxicological, and pharmacological investigations [29].

## 4. Historical and Cultural Uses of *O. glabra*

**Figure 3:**
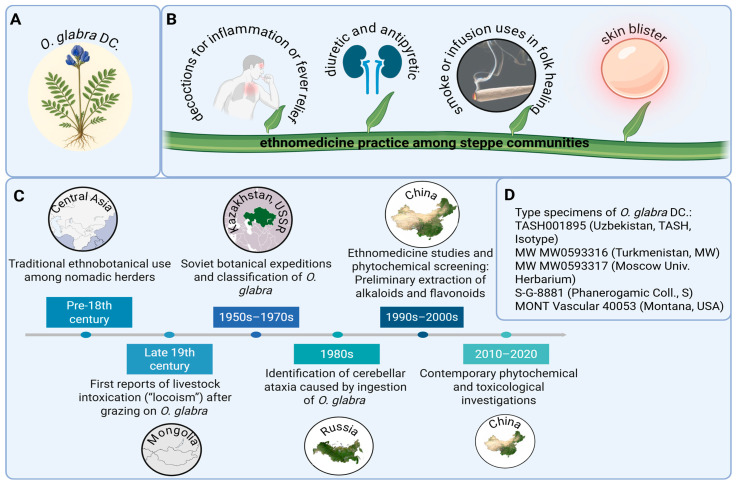
Historical and cultural uses of *O. glabra* across Central Asia: (**A**). Botanical illustration of *O. glabra*; (**B**). Ethnomedicinal practices among steppe communities, where the plant was traditionally used as a decoction or infusion for inflammation, fever, and diuretic purposes, and occasionally in smoke-based folk healing [6,14,24,26]; (**C**). Chronological overview of the documentation and regional studies on *O. glabra*, from pre-18th-century ethnobotanical use to 20th-century botanical expeditions and modern phytochemical investigations across Kazakhstan, Mongolia, Russia, and China [6,14,24]; (**D**). Type specimens and herbarium records of *O. glabra*, showing its verified taxonomic identity within the genus *Oxytropis* based on herbarium and occurrence data [30].

*O. glabra* has played a multifaceted role in the cultural and scientific heritage of Central Asia, reflecting its dual identity as both a traditional medicinal herb and a subject of botanical research. The species, widely distributed across Kazakhstan, Mongolia, and western China, has long been recognized by nomadic populations for its distinctive morphology and adaptive capacity to arid steppe environments [6,14,24]. As a member of the Fabaceae family, *O. glabra* (Figure 3A) occupies an important ecological niche in alpine meadows and semi-desert regions, where it was historically identified and cataloged in early botanical records. Its vernacular names, including the Kazakh “тықыp кeкipe,” point to its deep integration into local ethnobotanical traditions and folk medicine, where it was regarded as both a useful and potentially hazardous plant depending on the context of its use [14,24].

Among steppe communities, *O. glabra* held considerable ethnomedicinal value, particularly in the treatment of inflammatory and febrile conditions. Decoctions and infusions prepared from the aerial parts of the plant were traditionally administered to alleviate inflammation, fever, and urinary ailments, while smoke or infusion-based preparations were occasionally applied for skin disorders and ritual cleansing [6,14,24,26] (Figure 3B). Such practices illustrate the empirical understanding of plant-based therapy among pastoral societies and emphasize how indigenous medical knowledge developed in harmony with environmental observation. The persistence of these uses over centuries underscores the practical experience and cultural continuity embedded in nomadic healing traditions, in which plants such as *O. glabra* served both medicinal and symbolic functions within local health systems.

Historical records further indicate that the perception and study of *O. glabra* evolved in parallel with the expansion of regional botanical research. Ethnobotanical documentation dating from the pre-18th century gradually gave way to formal classification efforts during Soviet botanical expeditions in Kazakhstan and adjacent territories (1950s–1970s), followed by extensive Chinese ethnomedicinal and phytochemical studies in the late 20th century [6,14,24] (Figure 3C). These investigations isolated various alkaloids and flavonoids, bridging folk usage with modern scientific inquiry. Verified herbarium specimens collected from Uzbekistan, Turkmenistan, Russia, and the United States have since consolidated their taxonomic identity within the genus *Oxytropis*, confirming consistent morphological and geographic characteristics across reference collections [30] (Figure 3D). Collectively, these historical and cultural insights illustrate how *O. glabra* transitioned from a regionally known ethnomedicinal plant to a documented species of scientific and pharmacological relevance.

## 5. Phytochemistry

**Figure 4:**
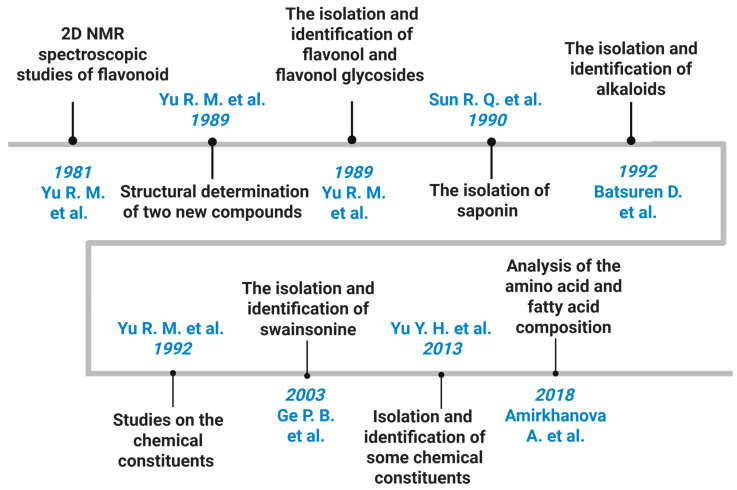
Chronological overview of phytochemical and analytical studies on *O. glabra* [16,17,18,19,20,21,22,23,24].

The phytochemical study of *O. glabra* has long attracted attention because of its toxicity to grazing animals and its historical use in traditional medicine across Central Asia. Early research aimed to explain livestock poisoning events in China, Mongolia, and Kazakhstan, which led to systematic investigations of the plant’s bioactive constituents. Over time, these studies established that *O. glabra* contains three dominant classes of secondary metabolites: quinolizidine alkaloids, flavonoids, and triterpenoid saponins, each contributing to its toxicological and pharmacological profile (Figure 4, Table 2) [16,17,18,19,20,21,22,23,24].

Yu Rongmin, Li Xian, and Zhu Tingru conducted one of the earliest detailed phytochemical investigations on *O. glabra*, identifying the major toxic alkaloid (−)-thermopsine, a quinolizidine compound with an LD_50_ of 89.98 mg/kg in mice, which confirmed its biological relevance in livestock poisoning. Their work provided the first chemical evidence linking the species to locoism and initiated subsequent research on its alkaloid composition [16].

Early phytochemical studies by the same group also identified a wide spectrum of flavonoids, including kaempferol and quercetin glycosides, isoquercitrin, several diglucosides, and isoflavanes [17,18,19]. These compounds form the core of the plant’s flavonoid profile and support its reported antioxidant and cytoprotective properties. Parallel studies documented triterpenoid saponins, including soyasapogenol B derivatives, and established the presence of complex glycosylated metabolites in the aerial parts [19].

Later investigations expanded the alkaloid profile of *O. glabra* and related *Oxytropis* species and confirmed numerous quinolizidine alkaloids such as anagyrine, sparteine, lupanine, *N*-formylcytisine, 13-hydroxysparteine, *N*-methylcytisine, baptifoline, and dictamnine [20]. These compounds are recognized contributors to neurotoxicity in grazing animals. Additional studies further identified diverse flavonol glycosides and nitrogen-containing metabolites, including quercetin-3-*O*-rutinoside, apigenin-7-oneohesperidoside, and kaempferol derivatives, demonstrating the phytochemical richness of the species [21,22,23].

More recent biochemical analyses quantified amino acids and fatty acids in *O. glabra*, revealing high levels of glutamic acid, aspartic acid, alanine, and linoleic and oleic acids, which provide complementary information on its metabolic composition [24].

**Figure 5:**
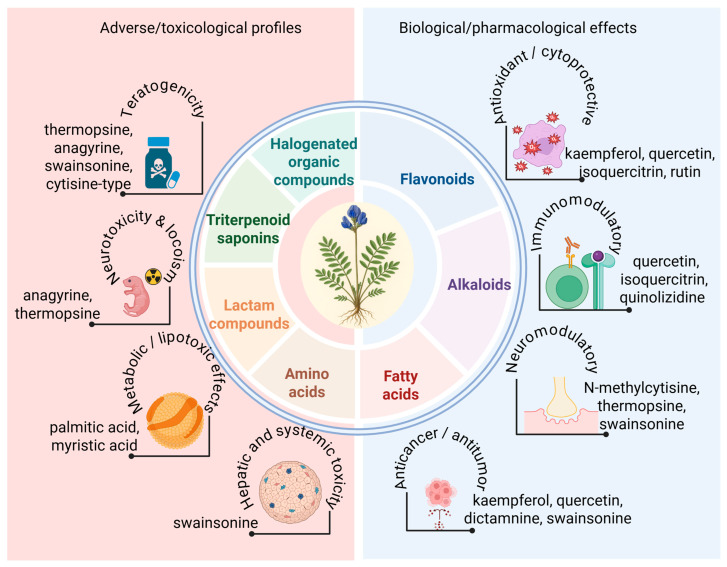
Major phytochemical classes of *O. glabra* and their associated pharmacological and toxicological effects.

Collectively, available data indicate that the phytochemistry of *O. glabra* is dominated by toxic quinolizidine alkaloids, bioactive flavonoids, and structurally complex triterpenoid saponins. However, gaps remain in understanding regional chemotype variation, biosynthetic pathways, and systematic pharmacological assessment beyond toxicity (Figure 5).

## 6. Bioactive Attributes of *O. glabra*

### 6.1. Alkaloids

**Figure 6:**
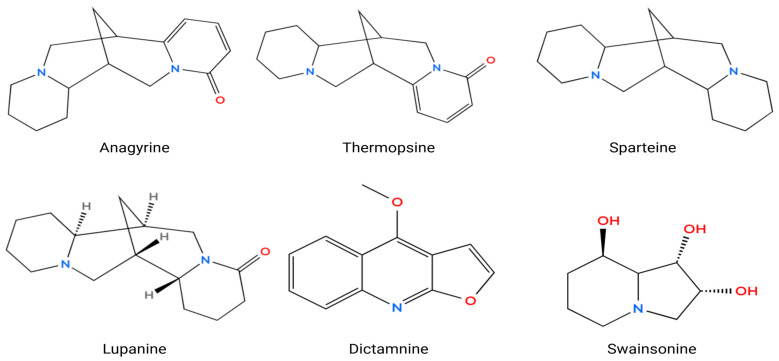
Chemical structures of major alkaloids identified in *O. glabra*. All structures were drawn using ChemDraw.

Anagyrine, a quinolizidine alkaloid found in various species of *Lupinus* and *Oxytropis*, has been thoroughly elucidated as a formidable teratogenic and neuroactive agent implicated in the etiology of congenital malformations in mammals (Figure 6) [42,45]. Preliminary toxicological investigations have identified anagyrine as the primary teratogen responsible for “crooked calf disease,” which is characterized by skeletal deformities, red cell aplasia, and vascular malformations in neonates subsequent to maternal consumption of lupine flora during gestation [42,45,47]. The teratogenic mechanism of anagyrine is linked to its interference with fetal neuromuscular development, primarily through the desensitization of nicotinic acetylcholine receptors (nAChRs), resulting in diminished fetal mobility and consequent musculoskeletal anomalies [43,49]. Green et al. substantiated that anagyrine engages directly with and desensitizes peripheral nAChRs independent of metabolic activation, indicating its prospective application as a biomarker for quinolizidine alkaloid teratogenicity in bovine species [43]. Quantitative pharmacokinetic analyses have further elucidated that serum concentrations of anagyrine attain peak levels within 2–12 h subsequent to ingestion, with elevated concentrations correlating with an increased severity of developmental defects [40]. Crystallographic analysis of anagyrine perchlorate has unveiled a stable tetracyclic framework characteristic of lupine alkaloids, substantiating its biological reactivity [44]. Methodologies in synthetic chemistry have adeptly replicated the stereochemistry of this molecule, facilitating intricate structure–activity relationship studies of analogous alkaloids such as thermopsine and cytisine [48]. Recent comprehensive reviews underscore that anagyrine constitutes approximately 4% of recognized quinolizidine alkaloids, highlighting its chemodiversity and pharmacological relevance within this chemical class [46]. In addition to its teratogenic profile, anagyrine exhibits measurable cytotoxic effects, with IC_50_ values of 27.3 ± 0.7 µg/mL in MCF-7 breast cancer cells and 30.2 ± 0.9 µg/mL in HEPG-2 liver cancer cells, as demonstrated in in vitro assays [80]. Furthermore, its ability to inhibit cytochrome P450 enzyme isoforms within the 2.5–50 µM range supports its classification as a multitarget bioactive agent [81]. Collectively, these findings affirm that anagyrine exerts teratogenic, neurotoxic, and cytotoxic actions via modulation of cholinergic receptors and disruption of neuromuscular signaling. It thus represents a structurally distinct and biologically potent natural toxin of concern in both veterinary and developmental toxicology [40,42,43,44,45,46,47,48,49,50].

Thermopsine, a quinolizidine alkaloid isolated from *Oxytropis* and *Thermopsis* species, exhibits both toxic and pharmacologically promising properties. Traditionally recognized for its neurotoxic and teratogenic effects in grazing animals, thermopsine acts as a modulator of nicotinic acetylcholine receptors (nAChRs), leading to neuromuscular impairment and fetal deformities when ingested in excessive amounts [51]. Beyond its toxicological significance, recent research has revealed notable antiviral activity of thermopsine derivatives. In particular, conjugates of (–)-cytisine and thermopsine were shown to inhibit the RNA-dependent RNA polymerase (RdRp) of SARS-CoV-1 and SARS-CoV-2, a key enzyme in viral RNA replication. Among these, the 1,3-dimethyluracil conjugate exhibited potent RdRp inhibition with an IC_50_ of 7.8 μM against SARS-CoV-2 and effectively suppressed viral replication, nucleocapsid protein expression, and subgenomic RNA synthesis with EC_50_ values of 0.12 μM for SARS-CoV-1 and 1.47 μM for SARS-CoV-2. Importantly, these potency values correspond to thermopsine–uracil conjugates rather than native thermopsine, indicating that structural modification significantly enhances antiviral efficacy [82]. This optimization potentially positions thermopsine derivatives as promising scaffolds for antiviral drug development. These findings collectively portray thermopsine as a structurally versatile alkaloid with dual biological relevance as a neuroactive toxin and as a potential therapeutic lead [51].

Sparteine, a naturally occurring quinolizidine alkaloid extracted from various species of the genera *Lupinus* and *Oxytropis*, demonstrates a range of pharmacological effects, particularly within the realm of the central nervous system (CNS). Empirical data suggest that sparteine exhibits anticonvulsant, neuromodulatory, and modest analgesic characteristics, all while preserving short-term memory, spatial learning, and typical behavioral responses. In experimental animal models, sparteine has shown a notable capacity to inhibit seizures elicited by maximal electro-stimulation and has delayed the onset of convulsive activity in mice subjected to pentylenetetrazole (PTZ). Additionally, it was observed to extend survival duration and mitigate the severity of seizures as well as mortality rates in rats administered PTZ or pilocarpine. Electrophysiological assessments indicated that sparteine diminishes both the amplitude and frequency of epileptiform discharges provoked by PTZ, pilocarpine, and kainic acid, thereby implying a direct attenuation of neuronal hyperexcitability. From a mechanistic standpoint, it is hypothesized that sparteine exerts its anticonvulsant effects via the activation of muscarinic acetylcholine receptor subtypes M2 and M4, which play a crucial role in modulating neuronal excitability and synaptic transmission, consequently stabilizing neuronal firing and thwarting seizure propagation [52].

Lupanine, a predominant quinolizidine alkaloid found in *Lupinus* and *Oxytropis* species, has demonstrated significant antidiabetic and insulinotropic properties through its action on pancreatic β-cells. Wiedemann et al. showed that lupanine enhances glucose-stimulated insulin secretion in INS-1E cells and isolated mouse islets at elevated glucose levels (≥15 mmol/L), while having no effect under low-glucose conditions, indicating a glucose-dependent mechanism that minimizes hypoglycemia risk. The compound upregulated Ins-1 gene expression, contributing to improved β-cell function and insulin synthesis. Mechanistically, lupanine modulates ATP-dependent potassium (KATP) channels, leading to membrane depolarization and increased Ca^2+^ action potential frequency, which in turn triggers insulin exocytosis. In electrophysiological studies, lupanine inhibited skeletal muscle sodium currents with an IC_50_ of approximately 1.2 mM, indicating relatively low potency toward voltage-gated Na^+^ channels and supporting its safety margin at concentrations effective for modulating β-cell KATP activity. In vivo, oral lupanine administration improved glucose tolerance and glycemic control in streptozotocin-induced diabetic rats without causing hypoglycemia. Collectively, these findings establish lupanine as a glucose-sensing insulin secretagogue and modulator of β-cell excitability, representing a promising alkaloid for managing type 2 diabetes mellitus through KATP channel regulation and insulin gene activation [53].

Dictamnine, a naturally occurring furoquinoline alkaloid predominantly extracted from the root bark of *Dictamnus dasycarpus* and also detected in *O. glabra*, demonstrates a wide array of pharmacological properties, notably its anticancer, anti-inflammatory, anti-pruritic, and antioxidative effects. Recent investigations have elucidated its robust anticancer efficacy through the direct inhibition of the receptor tyrosine kinase c-Met, resulting in the suppression of the PI3K/AKT/mTOR and MAPK signaling pathways, which serve as pivotal regulators of tumor cell proliferation and survival. Dictamnine has been shown to significantly impede the proliferation of c-Met-dependent lung adenocarcinoma cells (IC_50_ = 2.811 µM), induce cell-cycle arrest, and enhance susceptibility to EGFR-TKI agents such as gefitinib and osimertinib, thereby surmounting drug resistance in lung cancer paradigms [56]. In addition to its oncological implications, dictamnine manifests substantial anti-pruritic and anti-inflammatory properties in 2,4-dinitrofluorobenzene (DNFB)-induced atopic dermatitis models, wherein it mitigated chronic itching, diminished epidermal thickening, and downregulated MrgprA3/TRPA1 expression, which are critical mediators of histamine-independent itch signaling [56]. Moreover, its anti-colitic and antioxidative capabilities were demonstrated in dextran sulfate sodium (DSS)-induced ulcerative colitis murine models, where dictamnine alleviated intestinal inflammation, reinstated epithelial barrier integrity, and inhibited ferroptosis by activating the Nrf2–Gpx4 antioxidant defense pathway [57].

SW, an indolizidine alkaloid extracted from species of locoweed, presents a bifunctional pharmacological and toxicological profile, acting as both a robust antineoplastic immunomodulator and a neurotoxic autophagy regulator. Initially delineated for its anticancer efficacy, SW exhibited antimetastatic, antiproliferative, and immunomodulatory effects against both murine and human neoplasms, effectively curtailing primary tumor development and metastatic dissemination by augmenting immune-mediated cytotoxic responses [64]. Consistent with its antiproliferative potential, SW demonstrates dose-dependent cytostatic activity across multiple human cancer cell lines. In human gastric carcinoma SGC-7901 cells, SW inhibited proliferation with an IC_50_ of 0.84 μg/mL (approximately 2.8 μM) at 24 h, and complete growth suppression occurred at 6.2 μg/mL. These effects were associated with S-phase arrest, intracellular calcium overload, reduced expression of p53 and Bcl-2, and increased expression of c-myc, ultimately leading to apoptosis [83]. Similar findings were observed in HL-60 promyelocytic leukemia cells, where both standard and purified SW showed potent antileukemic activity with IC_50_ values of 6.96 μM and 9.50 μM at 48 h. These treatments resulted in substantial S-phase arrest, reaching 48.81 percent at 36 h and 60.72 percent at 48 h, which indicates inhibition of DNA synthesis and progression through the cell cycle [84]. Despite these therapeutic effects, subsequent investigations revealed that SW induces toxicity through disruption of autophagy and lysosomal pathways. Proteomic analyses demonstrated that SW impairs *O*-linked *N*-acetylglucosaminylation and inhibits the maturation of cathepsin D, which compromises lysosomal degradation and promotes cellular cytotoxicity [65]. Studies in mouse hippocampal neurons (HT22 cells) further showed that SW triggers vacuolar degeneration resulting from excessive autophagy through dysregulation of the PI3K/AKT/mTOR, ERK/mTOR, and p53/mTOR signaling pathways. Inhibition of autophagic degradation mitigates these cytopathological consequences [66]. Collectively, these findings confirm that SW exerts both therapeutic antiproliferative effects and significant neurotoxic potential through its coordinated actions on glycoprotein processing, lysosomal function, calcium signaling, and autophagy regulation.

### 6.2. Flavonoids

**Figure 7:**
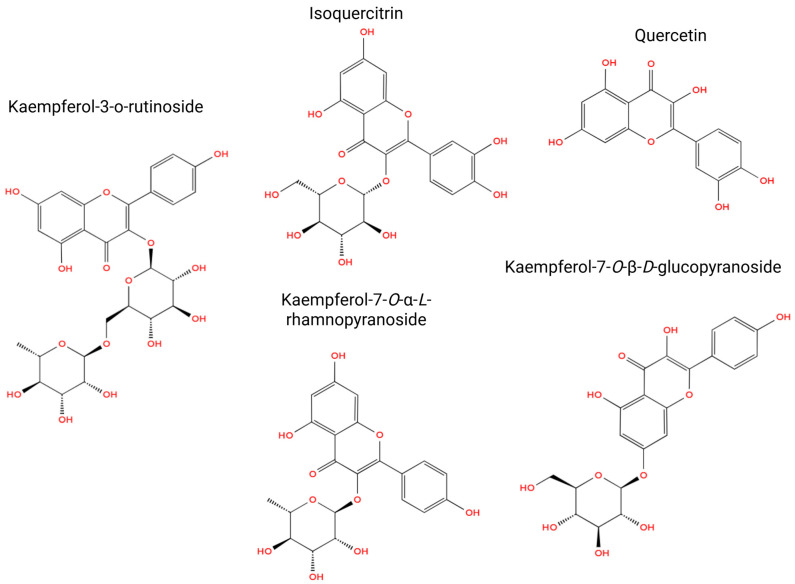
Chemical structures of major flavonoids identified in *O. glabra*. All structures were drawn using ChemDraw.

Kaempferol-3-*O*-rutinoside demonstrates a spectrum of biological activities with significant pharmacological implications (Figure 7). Recent investigations have elucidated its cardioprotective capabilities, as KR markedly enhanced cardiac function and diminished ventricular remodeling post-acute myocardial infarction by inhibiting the NF-κB/NLRP3/Caspase-1 signaling pathway, consequently mitigating myocardial fibrosis, apoptosis, and the production of inflammatory cytokines [35]. Supporting in vitro investigations corroborated its anti-inflammatory attributes, wherein KR inhibited nitric oxide synthesis and downregulated the expression of TNF-α, IL-6, iNOS, and COX-2 in LPS-stimulated RAW264.7 macrophages via a coordinated suppression of NF-κB and MAPK phosphorylation pathways [36]. In vivo studies further revealed that KR conferred hepatoprotective effects against CCl_4_-induced oxidative liver damage in murine models, reinstating serum total protein levels, diminishing AST, ALP, and MDA accumulation, and normalizing GSH, CAT, and SOD activities alongside enhanced histological morphology [37]. Moreover, KR exhibited significant α-glucosidase inhibitory activity, showing over eight-fold greater enzyme inhibition compared to acarbose, with synergistic enhancement evident when combined with kaempferol or quercetin [38]. In summary, these findings indicate that KR represents a multifaceted bioactive compound endowed with cardioprotective, hepatoprotective, anti-inflammatory, and antidiabetic properties, primarily mediated through the modulation of oxidative and inflammatory signaling pathways [35,36,37,38].

Isoquercitrin manifests a diverse array of pharmacological activities predominantly facilitated by its robust antioxidant and anti-inflammatory properties. The antioxidant efficacy of isoquercitrin is ascribed to its capacity to scavenge reactive oxygen and nitrogen species, including superoxide, hydroxyl, peroxyl radicals, and peroxynitrite, as well as to inhibit prooxidant enzymes such as xanthine oxidase and myeloperoxidase. Furthermore, isoquercitrin modulates oxidative stress responses in macrophages through the inhibition of p47phox phosphorylation, consequently obstructing the NADPH oxidase-mediated respiratory burst. In addition to its antioxidant properties, isoquercitrin exhibits considerable anti-inflammatory effects by decreasing the production of nitric oxide and prostaglandin E_2_, inhibiting the expression of iNOS and COX-2, and downregulating pro-inflammatory cytokines, including TNF-α and IL-6. It also manifests anti-angiogenic and anticancer properties by obstructing AP-1 activation, reducing MMP-9 expression, and impeding endothelial cell tube formation, which collectively diminishes tumor-associated angiogenesis. Moreover, isoquercitrin exhibits neuroprotective and cytoprotective functions by mitigating oxidative stress-induced apoptosis, fostering neurite outgrowth, and activating the SREBP-2 and Nrf2 signaling pathways that promote sterol synthesis and enhance antioxidant enzyme expression. Additional documented effects encompass the inhibition of α-glucosidase and α-amylase enzymes associated with glucose metabolism, the suppression of tyrosinase-mediated melanogenesis, and the modulation of xenobiotic-metabolizing enzymes such as CYP1A1 and CYP1B1 [39,40].

Beyond these well-established activities, recent studies have provided quantitative evidence of isoquercitrin’s anticancer potency, particularly in nasopharyngeal carcinoma models. Isoquercitrin reduced the viability of CNE1 and HNE1 cells with IC_50_ values of 392.45 μM and 411.38 μM, respectively, and induced ferroptotic cell death by elevating reactive oxygen species, increasing lipid peroxidation, suppressing GPX4 expression, and inhibiting AMPK and NF-κB p65 signaling. These effects diminished IL-1β production, restrained tumor growth in vivo, and were reversed by Ferrostatin-1 or alpha-lipoic acid, demonstrating pathway-specific ferroptosis regulation [85]. Isoquercitrin also displays antimicrobial potential through enzymatic inhibition of Streptococcus suis sortase A (Ss-SrtA), a virulence-associated transpeptidase. Biochemical assays revealed an IC_50_ of 100.0 ± 1.3 μM, indicating its ability to disrupt cell-surface protein anchoring and attenuate bacterial pathogenicity [86]. Collectively, these investigations underscore isoquercitrin as a multifunctional bioactive compound endowed with antioxidant, anti-inflammatory, neuroprotective, hepatoprotective, antidiabetic, anti-angiogenic, chemopreventive, and newly recognized ferroptosis-inducing and antimicrobial properties across a multitude of biological systems.

Quercetin, a widely distributed flavonol found in a multitude of therapeutic plants, exerts an extensive array of metabolic, antioxidative, and anti-inflammatory effects, with substantial evidence supporting its efficacy in experimental models of diabetes, dyslipidemia, hypertension, and obesity. Investigations involving streptozotocin (STZ)-induced diabetic rats revealed its antihyperglycemic and pancreatic-protective properties, as the administration of quercetin (10–15 mg/kg for 10–12 weeks) markedly diminished blood glucose, triglycerides (TG), and total cholesterol (TC), whilst augmenting hexokinase, glucokinase, and antioxidant enzyme activities, thereby enhancing pancreatic islet quantity and insulin secretion [58]. These observed effects were correlated with the activation of hepatic SIRT1 and Akt, indicating potential insulin-sensitizing and glucose metabolism-modulating attributes. Quercetin also mitigated oxidative stress and inflammation, leading to a reduction in the levels of TNF-α, C-reactive protein (CRP), and NF-κB activity, while simultaneously upregulating antioxidant defenses and sirtuin signaling in diabetic models. In contexts of hyperlipidemia and hypertension, quercetin exhibited hypolipidemic and vasoprotective effects by restoring serum cholesterol, TG, LDL, and HDL levels to normal ranges, inhibiting lipid peroxidation, and reinstating NO-mediated endothelial function in both diet-induced and L-NAME-induced hypertension models. Its antioxidative and nitric oxide-enhancing characteristics contributed to diminished vascular injury and hepatic enzyme activity, thereby signifying hepatic protection and enhanced lipid metabolism. Furthermore, quercetin demonstrated anti-obesity effects by decreasing adiposity and inflammatory cytokine levels while augmenting adiponectin concentrations and activating the AMPKα1/SIRT1 signaling pathway, which resulted in the regulation of GLUT4 and improved glucose utilization. In addition to these metabolic and anti-inflammatory actions, quercetin displays potent cytoprotective and anticancer effects supported by quantitative IC_50_ data. In non-tumorigenic human thyroid Nthy-ori-3-1 cells, cadmium chloride reduced viability with an IC_50_ of approximately 10 μM, whereas quercetin supplementation restored ERK phosphorylation, attenuated reactive oxygen species, reduced lipid peroxidation, and decreased GRP78 expression, indicating protection against cadmium-induced oxidative and endoplasmic reticulum stress [87]. Quercetin also acts as a strong enzymatic inhibitor of xanthine oxidase, with IC_50_ values of 2.74 × 10^−6^ mol L^−1^ for uric acid formation and 2.90 × 10^−6^ mol L^−1^ for superoxide generation through mixed-type inhibition mediated by high-affinity binding within the FAD domain [88]. In cancer models, quercetin inhibited proliferation of MCF-7 breast cancer cells with an IC_50_ of 37 μM, inducing G1 arrest and apoptosis through suppression of Twist, Cyclin D1, p21, and phospho-p38 MAPK, whereas MDA-MB-231 cells showed limited sensitivity even at 100 μM [89]. Collectively, these findings show that quercetin demonstrates concentration-dependent cytoprotection, enzymatic inhibition, and anticancer activity through modulation of oxidative stress responses, cell-cycle regulators, and transcriptional pathways.

Kaempferol-7-*O*-α-*L*-rhamnopyranoside (KR), a glycosylated flavonoid isolated from *Nephelium lappaceum* and various other medicinal flora, manifests a plethora of pharmacological and biochemical attributes pertinent to cardiovascular, dermatological, and biochemical modulation. Functionally, KR exhibits a significant vasodilatory effect via endothelium-dependent pathways, wherein it augments eNOS phosphorylation and nitric oxide (NO) synthesis, thereby activating the NO–cGMP–PKG signaling cascade and facilitating the relaxation of vascular smooth muscle. This phenomenon was accompanied by a reduction in phosphorylated MLC and PKC levels, thereby substantiating its ability to induce vascular relaxation and contribute to antihypertensive efficacy without exhibiting cytotoxicity [59]. In addition to its cardiovascular protective effects, KR demonstrates notable melanin-inhibitory and tyrosinase-suppressing activities, surpassing the efficacy of other kaempferol glycosides in B16F10 melanoma cells. When synergistically combined with arbutin, KR manifests an enhanced whitening effect and bolsters the stability of cosmetic formulations, preserving over 90% of active constituents under accelerated storage conditions, thereby indicating its potential applications in skin-lightening and dermatocosmetic products [60]. Furthermore, KR exhibits strong and specific interactions with human serum albumin (HSA) through hydrogen bonding, van der Waals forces, and electrostatic attractions, thereby forming a stable exothermic complex that induces conformational alterations in the secondary structure of HSA, highlighting its significance for drug transport, bioavailability, and potential modulation of diabetic complications [61].

Kaempferol-3-*O*-β-*D*-glucopyranoside is a flavonol 3-*O*-glucoside that has been extensively investigated for its broad spectrum of biological activities. Accumulating experimental evidence demonstrates pronounced hepatoprotective effects, which are largely attributed to its strong antioxidant capacity and ability to suppress inflammatory signaling cascades. At the molecular level, this compound modulates bile acid homeostasis and lipid metabolism through regulation of the farnesoid X receptor (FXR), while simultaneously inhibiting the TLR4/MYD88/JNK signaling pathway, thereby attenuating hepatic inflammation and oxidative stress. Beyond hepatoprotection, kaempferol-3-*O*-β-*D*-glucopyranoside exhibits immunomodulatory activity by regulating cytokine production and immune cell activation, contributing to its anti-inflammatory profile. In addition, multiple in vitro and in vivo studies have reported antitumor and antileukemic effects, mediated through the induction of apoptosis, suppression of cell proliferation, and interference with oncogenic signaling pathways [62,63]. Collectively, these findings position kaempferol-3-*O*-β-*D*-glucopyranoside as a multifunctional bioactive flavonoid with significant therapeutic potential, particularly in the context of liver disorders, inflammatory diseases, and cancer.

### 6.3. Lactam Compounds

Caprolactam, a cyclic amide monomer pivotal for the biosynthesis of nylon-6, has garnered significant attention as a target metabolite within the realms of biotechnology and synthetic biology. Recent developments have yielded an artificially constructed caprolactam-specific riboswitch, designed as an intracellular metabolite biosensor to enhance high-throughput metabolic engineering and optimize metabolic pathways in recombinant microbial strains. By employing a coupled in vitro–in vivo selection methodology, investigators developed a heterogeneous RNA aptamer library via Systematic Evolution of Ligands by Exponential Enrichment (SELEX), thereby identifying riboswitch variants that displayed robust and selective binding affinity for caprolactam. The refined riboswitch demonstrated a 3.36-fold increase in activation in the presence of 50 mM caprolactam and effectively differentiated it from structurally analogous compounds such as valerolactam, thereby affirming its exceptional specificity and selectivity. Functionally, the riboswitch facilitated caprolactam-dependent gene expression and growth regulation, thus serving as a potent instrument for intracellular metabolite sensing, dynamic modulation of metabolic fluxes, and the screening of enhanced caprolactam-producing strains, which signifies a noteworthy advancement towards the biobased production of nylon-6 [67,68].

### 6.4. Amino Acids

The amino acid composition elucidated in *O. glabra* [24] exemplifies a well-balanced profile of both essential and non-essential components that facilitate metabolic regulation, structural integrity, and physiological homeostasis. Nonpolar aliphatic amino acids, including alanine, glycine, leucine, isoleucine, and valine, play a pivotal role in energy metabolism and muscle physiology. Alanine is involved in glucose metabolism via the alanine–glucose cycle, whereas glycine functions as a neurotransmitter and is instrumental in collagen synthesis and antioxidant defense mechanisms. Leucine, isoleucine, and valine are known to stimulate muscle protein synthesis, modulate glucose uptake, and assist in maintaining nitrogen balance. Acidic amino acids such as glutamic acid and aspartic acid are crucially engaged in neurotransmission and the urea cycle, while polar amino acids, including threonine, serine, and methionine, are significant contributors to protein synthesis, lipid metabolism, and antioxidant pathways. Proline and hydroxyproline augment the strength of connective tissues and facilitate wound healing, and cystine plays a vital role in disulfide bond formation and the structural stability of proteins [69,70,71,72,73,74].

Essential and specialized amino acids such as phenylalanine, tyrosine, histidine, ornithine, arginine, lysine, and tryptophan further underscore the biochemical diversity inherent in the amino acid composition of *O. glabra*. Phenylalanine and tyrosine serve as precursors for the synthesis of neurotransmitters and hormones, while tryptophan is integral to the biosynthesis of serotonin, melatonin, and niacin, thereby influencing mood and sleep regulation. Histidine, arginine, and lysine are essential for maintaining immune and vascular functions, with arginine significantly enhancing the production of nitric oxide and promoting vasodilation. Ornithine acts as an intermediate in the urea cycle, facilitating cellular detoxification and regeneration processes. Collectively, these amino acids underpin critical metabolic processes, bolster antioxidant defense, and contribute to the structural and signaling mechanisms that are vital for physiological stability and adaptive responses within biological systems [69,70,71,72,73,74].

### 6.5. Fatty Acids

The fatty acid composition characterized in *O. glabra* [24] reveals a meticulously balanced arrangement of saturated, monounsaturated, and polyunsaturated fatty acids that collectively facilitate cellular architecture, metabolic regulation, and physiological safeguarding. Saturated fatty acids, including myristic, pentadecanoic, palmitic, and stearic acids, confer structural resilience to cellular membranes and act as energy reserves. Myristic acid manifests antimicrobial and surfactant characteristics while participating in lipid anchoring and protein alteration; in contrast, pentadecanoic acid, an odd-chain fatty acid, serves as a biomarker indicative of dairy fat consumption and elicits insulin-sensitizing and cardioprotective responses. Palmitic acid serves as a predominant structural lipid and a precursor for signaling molecules, although its excessive accumulation may precipitate lipotoxic and pro-inflammatory consequences. Stearic acid, classified as a long-chain saturated fatty acid, aids in membrane stabilization and energy sequestration and is regarded as neutral with respect to plasma cholesterol concentrations. Collectively, these saturated fatty acids uphold membrane integrity and energy metabolism while exerting influence over metabolic and inflammatory pathways [75,76,77,78,79].

The monounsaturated and polyunsaturated fatty acids identified in *O. glabra* further augment its nutritional and therapeutic significance. Palmitoleic acid, characterized as a monounsaturated lipid with lipokine functionality, enhances insulin sensitivity and modulates hepatic lipid metabolism. Oleic acid, an ω-9 monounsaturated fatty acid, is particularly recognized for its anti-inflammatory, antioxidant, and cardioprotective attributes, which contribute to enhanced lipid profiles and membrane fluidity. Among the polyunsaturated fatty acids, linoleic and linolenic acids serve critical functions in sustaining physiological homeostasis. Linoleic acid, an ω-6 fatty acid, acts as a precursor to arachidonic acid while supporting skin barrier integrity and inflammatory modulation. Linolenic acid, an ω-3 fatty acid, displays neuroprotective, cardiovascular, and anti-inflammatory properties as a biosynthetic precursor of EPA and DHA. Collectively, these fatty acids constitute fundamental components that underpin structural, metabolic, and protective processes within biological systems [75,76,77,78,79].

## 7. Toxicity

The deleterious effects of *O. glabra* can be predominantly ascribed to its alkaloidal components, with particular emphasis on SW, an indolizidine alkaloid identified as the primary toxic agent implicated in locoism and chronic toxicity in herbivorous fauna. Affected agricultural animals manifest a progressive array of neurological manifestations, encompassing ataxia, head tremors, dyskinesia, and behavioral anomalies, frequently accompanied by reproductive issues such as infertility and abortion, alongside a general decline in metabolic health. SW functions as a robust and selective inhibitor of α-mannosidase (α-Man), engaging in competitive binding to its active site, attributable to its semi-chair conformation, which bears resemblance to the substrate of the enzyme. This inhibition precipitates a disruption in the *N*-glycosylation pathway by obstructing the maturation of mannose-enriched oligosaccharides, thereby altering the synthesis and trafficking of glycoproteins, which culminates in the accumulation of intracellular oligosaccharides and extensive vacuolar degeneration within neural and parenchymal cells. In conjunction with α-Man inhibition, SW also impedes the enzymatic activities of *N*-acetylglucosaminyltransferase I and II, consequently impairing the structural integrity of the *N*-glycan sugar chain throughout the processing of glycoproteins, with downstream repercussions on cellular adhesion, migration, and differentiation. The aggregation of these biochemical disruptions yields pronounced vacuolar degeneration in cerebellar Purkinje neurons and parenchymal tissues, with neural injuries remaining irreparable even subsequent to the termination of exposure [66,67,90,91,92,93,94,95,96,97,98,99,100,101].

Experimental in vitro assays have elucidated that SW induces cytotoxicity via multiple cellular death pathways that encompass both apoptosis and autophagy. In neuronal models, SW was found to significantly upregulate the expression of caspases-3, -8, and -12 while simultaneously increasing intracellular calcium levels, thereby implicating death receptor and endoplasmic reticulum (ER) stress-mediated apoptotic mechanisms [90,91]. In goat trophoblasts, SW facilitated the translocation of Bax to mitochondria, the release of cytochrome c, and the subsequent activation of caspase-9 and caspase-3, culminating in poly(ADP-ribose) polymerase (PARP) cleavage and apoptosis through the mitochondrial pathway [93]. Analogous findings in renal tubular epithelial cells revealed that SW elevated intracellular Ca^2+^, induced ER expansion, caused proteasomal damage, and led to vacuolization through the MAPK and ER stress pathways [94]. Furthermore, SW was demonstrated to initiate autophagy via the inhibition of PI3K/AKT/mTOR signaling, accompanied by the activation of ERK/mTOR and p53/mTOR pathways, while lysosomal dysfunction obstructed autophagic degradation [85,86,87]. Proteomic investigations revealed that SW modifies the O-GlcNAcylation of cathepsin D (CTSD), leading to a reduction in the formation of its mature isoform (m-CTSD), which impairs lysosomal function and promotes cytotoxicity [66]. Collectively, these findings delineate a mechanistic framework in which SW-induced autophagy and apoptosis converge through the disruption of glycosylation and lysosomal homeostasis.

In vivo evaluations have further corroborated the chronic toxicity of SW, characterized by cumulative neurological and reproductive impairments. Prolonged consumption of *O. glabra* results in extensive vacuolar degeneration across both neural and visceral tissues, with the cerebellar Purkinje cells exhibiting the most pronounced lesions [96]. While damage to non-neural organs tends to reverse upon cessation of exposure, neuronal degeneration persists in a permanent state [97]. Studies assessing reproductive toxicity indicate that SW prolongs the estrous cycle, decreases ovulatory frequency, and impairs fertility and offspring viability by adversely affecting the glycosylation of gonadotropin glycoproteins, which leads to disordered steroidogenesis and hormonal imbalance during gestation [98,99,100,101]. Moreover, hepatotoxicity and systemic inflammation have been associated with SW-induced alterations in bile acid metabolism and intestinal microbiota composition, which contribute to hepatic injury and metabolic dysregulation [93]. In addition to SW, minor alkaloids such as 2,6,6-tetramethyl-4-piperidone (TMPD) also demonstrate α-Man inhibitory activity, thereby reinforcing the role of piperidine and quinolizidine derivatives in the overarching toxicity of *O. glabra*. Collectively, these findings establish SW as the primary determinant of *O. glabra* toxicity, mediating a complex cascade of glycoprotein processing inhibition, ER stress, apoptosis, and autophagy that culminates in chronic neurological and systemic pathologies in animal models [97,98,99,100,101].

## 8. Integrative Perspective: Toxicological Risks Versus Pharmacological Opportunities

The available evidence positions *O. glabra* as a phytochemically dualistic species in which toxic and therapeutically relevant metabolites coexist within the same chemical framework, but the toxicological dimension is currently better documented than any potential clinical benefit. The dominant quinolizidine and indolizidine alkaloids, including anagyrine, thermopsine, sparteine, lupanine, and swainsonine, are robustly linked to teratogenic, neurotoxic, and chronic locoism syndromes in grazing livestock. These toxicities arise from discrete molecular mechanisms such as nicotinic acetylcholine receptor modulation [40,42,43,44,45,46,47,48,49,50,51], α-mannosidase inhibition [64,65,66,90,91,92,93,94,95,96,97,98,99,100,101], and disruption of glycoprotein processing and lysosomal function, which together drive vacuolar degeneration and cellular dysfunction. In vivo studies consistently demonstrate irreversible neuronal vacuolization, reproductive impairment, and systemic metabolic alterations after prolonged exposure, yet dose–response relationships and species differences remain incompletely defined. As a result, uncontrolled ingestion of *O. glabra* clearly represents a major veterinary hazard, while the implications for human exposure through traditional remedies or accidental intake remain largely speculative and insufficiently quantified.

In contrast to these toxic liabilities, several metabolites of *O. glabra* exhibit pharmacological activities in reductionist experimental systems, although most data derive from isolated compounds or non-*Oxytropis* matrices and may not be directly extrapolable to whole-plant use. Lupanine enhances glucose-dependent insulin secretion without inducing hypoglycemia in rodent models [53], and thermopsine-derived conjugates inhibit coronavirus RNA-dependent RNA polymerase at low micromolar concentrations [51,82]. Swainsonine and dictamnine show antiproliferative, antimetastatic, and immunomodulatory actions in selected tumor models [55,56,64,65,66,83,84], but these same alkaloids also contribute to neurotoxicity and systemic toxicity at overlapping concentration ranges. The flavonoid fraction, including kaempferol, quercetin, isoquercitrin, kaempferol-3-O-rutinoside, and KGR, displays antioxidant, anti-inflammatory, cardioprotective, hepatoprotective, and chemopreventive effects [35,36,37,38,39,40,41,58,59,60,61,62,63]. However, most of these activities are demonstrated under in vitro or acute in vivo conditions that do not capture the chronic co-exposure to toxic alkaloids typical of locoism. Therefore, although several constituents possess drug-like features, their translational value remains uncertain in the absence of rigorous safety pharmacology and context-appropriate dosing studies.

This duality underscores the need to define not only therapeutic windows and structure–activity relationships for individual metabolites but also the net risk–benefit balance of realistic exposure scenarios. At present, critical knowledge gaps include the lack of comparative studies linking in vitro IC_50_ or EC_50_ values to achievable in vivo concentrations in target organs, limited information on regional chemotype variation and how it shifts the ratio between toxic alkaloids and protective flavonoids, and the absence of standardized *O. glabra* extracts with reproducible profiles. Moreover, available pharmacokinetic and bioavailability data are sparse or derived from other plant sources, which prevents robust estimation of safety margins for *O. glabra* preparations. Without these data, any extrapolation from experimental models to traditional or prospective clinical use remains speculative and potentially misleading.

Future research should therefore move beyond cataloging bioactivities and focus on integrative toxicological and pharmacological assessment. Priority directions include systematic in vivo dose–response and exposure–response studies, careful pharmacokinetic characterization of key alkaloids and flavonoids, and medicinal chemistry programs aiming to separate beneficial targets from off-target toxicities, for example, by modifying thermopsine or swainsonine scaffolds to attenuate α-mannosidase inhibition while preserving antiviral or antitumor activity. Parallel mapping of biosynthetic pathways and environmental drivers of chemotype variability is needed to understand when and where highly toxic chemotypes arise and whether safer profiles can be obtained. Only through this combined approach will it be possible to determine whether *O. glabra* should remain classified primarily as a hazardous locoweed or whether selected, chemically optimized derivatives can advance toward preclinical or clinical development as antiviral, antidiabetic, or anticancer candidates.

## 9. Conclusions and Future Perspectives

The available evidence positions *O. glabra* as a chemically rich and ecologically resilient species with a well-established taxonomic identity and a pronounced dual biological profile. While the plant is widely recognized in veterinary medicine as a toxic locoweed, its phytochemical composition also includes metabolites with documented pharmacological potential. Quinolizidine and indolizidine alkaloids are primarily responsible for toxicity, whereas flavonoids and related compounds contribute antioxidant, anti-inflammatory, hepatoprotective, and cytoprotective activities. This coexistence of toxic and bioactive constituents underscores the need to view *O. glabra* not solely as a rangeland hazard, but also as a potential source of lead compounds when approached under controlled and well-defined conditions.

At the same time, the toxicological risk associated with *O. glabra* is unequivocal. Swainsonine remains the dominant driver of chronic poisoning in livestock, and its effects explain the persistent neurological and reproductive damage observed in exposed animals. These risks represent a critical barrier to therapeutic exploitation and highlight the necessity of defining compound-specific toxicity thresholds, standardizing extracts, and separating toxic from potentially beneficial constituents prior to any translational application.

Future research should focus on several clearly defined directions. First, comprehensive and modern phytochemical profiling using high-resolution analytical platforms is required to update and expand existing metabolite inventories and to resolve chemotype variability across geographic regions and developmental stages. Second, rigorous pharmacokinetic, bioavailability, and safety evaluations are needed to validate reported biological activities beyond in vitro and short-term in vivo models. Third, applied ecological and veterinary strategies, including phytochemical monitoring of natural populations, should be integrated to mitigate livestock exposure in high-risk environments. Collectively, these approaches will enable a balanced reassessment of *O. glabra* as both a toxic species of veterinary concern and a controlled natural resource with potential relevance to drug discovery.

## Figures and Tables

**Table 1:** The number of searches for each keyword.

Keywords	Searches		
	Google Scholar	PubMed	Scopus
*Oxytropis glabra*	1670	9	55
*Oxytropis glabra* compounds	170	—	5
*Oxytropis glabra* phytochemicals	70	1	2
*Oxytropis glabra* Pharmacological	55	—	—
*Oxytropis glabra* toxicity	173	—	—
*Oxytropis glabra* traditional uses	381	—	1

“—” means not found.

**Table 2:** Phytochemicals, primary metabolites, and reported pharmacological activities identified from *O. glabra*.

No.	Compound Name	Chemical Class	Subclass	Pharmacological Activity
1	Thermopsine [16]	Alkaloid	Quinolizidine alkaloid	—
2	1,1,1,7,7,7-Hexachloro-2,6-dihydroxyheptan-4-one [17]	Halogenated organic compound	Chlorinated aliphatic ketone	—
3	Kaempferol 7-*O*-rhamnoside [17,18]	Flavonoid	Flavonol glycoside	immune checkpoint inhibition (PD-1/PD-L1 blockade) [31], antioxidant [32], anti-inflammatory [33], and antitumor activities [34]
4	Kaempferol 3-*O*-diglucoside [18]	Flavonoid	Flavonol diglycoside	—
5	Kaempferol 3-*O*-rutinoside [18]	Flavonoid	Flavonol glycoside (rutinoside)	cardioprotective, anti-inflammatory, hepatoprotective, antioxidant, antidiabetic, antifibrotic, antiapoptotic, and α-glucosidase inhibitory properties [35,36,37,38].
6	Kaempferol 3-*O*-glucoside-7-O-glucoside [18]	Flavonoid	Flavonol diglycoside	—
7	Isoquercitrin [18]	Flavonoid	Flavonol 3-*O*-glucoside	antioxidant, anti-inflammatory, anti-angiogenic, anticancer, neuroprotective, hepatoprotective, antidiabetic, cytoprotective, anti-apoptotic, anti-melanogenic, chemopreventive, enzyme inhibitory (α-glucosidase, α-amylase, tyrosinase, CYP1A1, CYP1B1) [39,40,41]
8	3′,7-Dihydroxy-2′,4′-dimethoxyisoflavane [18]	Isoflavonoid	Isoflavane-type compound	—
9	3-*O*-[α-L-rhamnopyranosyl-(1→2)-β-Oglucopyranosyl(1→4)-β-*D*-glucuronopyranosyl]-soyasapogenol B [19]	Triterpenoid saponin	Oleanane-type saponin	—
10	Anagyrine [20]	Alkaloid	Quinolizidine alkaloid	teratogenic, neurotoxic, and cytotoxic [40,42,43,44,45,46,47,48,49,50]
11	Thermopsine [20]	Alkaloid	Quinolizidine alkaloid	neurotoxic, teratogenic, nAChR-modulating, antiviral (SARS-CoV-1 and SARS-CoV-2 RdRp inhibitory) [51]
12	Sparteine [20]	Alkaloid	Quinolizidine alkaloid	anticonvulsant, neuromodulatory, antiepileptic, mild analgesic, neuroprotective, muscarinic receptor (M2/M4) agonist, seizure-suppressive [52]
13	Lupanine [20]	Alkaloid	Quinolizidine alkaloid	antidiabetic, insulinotropic, glucose homeostasis regulator, KATP channel inhibitor, β-cell depolarizing agent, insulin gene (Ins-1) upregulator, non-hypoglycemic [53]
14	*N*-Formylcytisine [20]	Alkaloid	Quinolizidine alkaloid (cytisine-type)	—
15	13-Hydroxysparteine [20]	Alkaloid	Quinolizidine alkaloid (hydroxylated)	—
16	*N*-Methylcytisine [20]	Alkaloid	Quinolizidine alkaloid (cytisine-type)	anti-inflammatory, antiviral (anti-dengue), pharmacokinetically bioavailable, anti-colitic, selective nicotinic acetylcholine receptor (nAChR) ligand, cholinergic agonist, neuroactive, behavior-modulating, cytotoxicity-measurable by UPLC–MS/MS [54]
17	Baptifoline [20]	Alkaloid	Quinolizidine alkaloid	—
18	Dictamnine [20]	Alkaloid	Furoquinoline alkaloid	anticancer, c-Met inhibitor, PI3K/AKT/mTOR and MAPK pathway suppressor, anti-inflammatory, anti-pruritic, anti-colitic, antioxidant, ferroptosis inhibitor, Nrf2–Gpx4 activator, EGFR-TKI sensitizer [55,56,57]
19	Myricitin 3-*O*-glucoside [21]	Flavonoid	Flavonol 3-*O*-glucoside	—
20	3-*O*-[α-L-rhamnopyranosyl-(1→3)-β-*D*-glucopyranosyl(1→6)-β-*D*-glucuronopyranosyl]-soyasapogenol B [21]	Triterpenoid saponin	Oleanane-type saponin	—
21	Quercetin [21]	Flavonoid	Flavonol aglycone	antihyperglycemic, hypolipidemic, hypotensive, anti-obesity, antioxidant, anti-inflammatory, hepatoprotective, insulin-sensitizing, AMPK/SIRT1 activator, GLUT4 regulator [58]
22	Kaempferol [21]	Flavonoid	Flavonol aglycone	immune checkpoint inhibition (PD-1/PD-L1 blockade) [30], antioxidant 31], anti-inflammatory [32], and antitumor activities [33]
23	Kaempferol-7-*O*-α-L-rhamnopyranoside [21]	Flavonoid	Flavonol glycoside	vasodilatory, antihypertensive, eNOS activator, NO–cGMP–PKG pathway modulator, anti-melanogenic, tyrosinase inhibitor, cosmetic whitening agent, HSA-binding bioactive, antioxidant, anti-inflammatory [59,60,61]
24	Kaempferol-3-*O*-β-*D*-glucopyranoside [21]	Flavonoid	Flavonol 3-*O*-glucoside	hepatoprotective, antioxidant, anti-inflammatory, immunomodulatory, FXR–TLR4/MYD88/JNK pathway inhibitor, bile acid synthesis regulator, antitumor, antileukemic [62,63]
25	Kaempferol-3-*O*-β-*D*-glucopyranosyl(1→2)-β-*D*-glucopyranoside [21]	Flavonoid	Flavonol diglycoside	—
26	Kaempferol-3-*O*-β-*D*-glucopyranosyl-7-*O*-β-*D*-glucopyranoside [21]	Flavonoid	Flavonol diglycoside	—
27	Quercetin-3-*O*-β-*D*-glucopyranoside [21]	Flavonoid	Flavonol 3-*O*-glucoside	—
28	Myricetin-3-*O*-β-*D*-glucopyranoside [21]	Flavonoid	Flavonol 3-*O*-glucoside	—
29	3-*O*-[α-*L*-rhamnopyranosyl(1→3)-β-*D*-glucopyranosyl(1→6)-β-*D*-glucuronopyranosyl]-soyasapogenol B [21]	Triterpenoid saponin	Oleanane-type saponin	—
30	3-*O*-[β-*D*-glucopyranosyl(1→2)-β-*D*-glucuronopyranosyl]-azukisapogenol amide [21]	Triterpenoid saponin	Oleanane-type amide saponin	—
31	Swainsonine (SW) [22]	Alkaloid	Indolizidine alkaloid	antineoplastic, immunomodulatory, antimetastatic, antiproliferative, autophagy modulator, lysosomal inhibitor, O-GlcNAcylation regulator, CTSD maturation inhibitor, neurotoxic, mTOR/PI3K/AKT/ERK/p53 pathway regulator [64,65,66]
32	Caprolactam [23]	Lactam compound	Cyclic amide (ε-caprolactam type)	metabolite biosensor, riboswitch specificity, SELEX-based aptamer selection, high-throughput screening tool, metabolic engineering aid, caprolactam-responsive regulator, nylon-6 bioproduction enhancer [67,68]
33	Quercetin-3-*O*-β-*D*-rutinoside [23]	Flavonoid	Flavonol glycoside (rutinoside; rutin)	—
34	Apigenin-7-Oneohesperidoside,3 [23]	Flavonoid	Flavone glycoside	—
35	Kaempeferol-3-*O*-β-*D*-glucopyranoside-7-*O*-β-*D*-glucopyranoside [23]	Flavonoid	Flavonol diglycoside	—
36	Alanine [24]	Amino acid	Nonpolar, aliphatic	supports glucose metabolism and energy production through the alanine–glucose cycle [69,70,71,72,73,74]
37	Glycine [24]	Amino acid	Nonpolar, aliphatic	functions as a neurotransmitter and contributes to collagen synthesis and antioxidant defense [69,70,71,72,73,74]
38	Leucine [24]	Amino acid	Nonpolar, aliphatic	stimulates muscle protein synthesis via mTOR signaling and promotes tissue repair [69,70,71,72,73,74]
39	Isoleucine [24]	Amino acid	Nonpolar, aliphatic	regulates glucose uptake, hemoglobin formation, and muscle recovery [69,70,71,72,73,74]
40	Valine [24]	Amino acid	Nonpolar, aliphatic	aids muscle growth, nitrogen balance, and endurance [69,70,71,72,73,74]
41	Glutamic acid [24]	Amino acid	Acidic (negatively charged)	acts as an excitatory neurotransmitter and precursor of GABA [69,70,71,72,73,74]
42	Threonine [24]	Amino acid	Polar, uncharged	maintains gut integrity and supports protein and mucin synthesis [69,70,71,72,73,74]
43	Proline [24]	Amino acid	Nonpolar, cyclic (imino acid)	strengthens collagen structure and promotes wound healing [69,70,71,72,73,74]
44	Methionine [24]	Amino acid	Nonpolar, sulfur-containing	provides methyl groups for DNA methylation and supports antioxidant synthesis [69,70,71,72,73,74]
45	Serine [24]	Amino acid	Polar, uncharged	participates in nucleotide, phospholipid, and amino acid biosynthesis [69,70,71,72,73,74]
46	Aspartic acid [24]	Amino acid	Acidic (negatively charged)	supports urea cycle, energy metabolism, and hormone regulation [69,70,71,72,73,74]
47	Cystine [24]	Amino acid	Sulfur-containing (derived from cysteine)	supports urea cycle, energy metabolism, and hormone regulation [69,70,71,72,73,74]
48	Oxyproline (Hydroxyproline) [24]	Amino acid	Modified imino acid	maintains collagen stability and tissue strength [69,70,71,72,73,74]
49	Phenylalanine [24]	Amino acid	Aromatic, nonpolar	precursor for tyrosine and catecholamine neurotransmitters [69,70,71,72,73,74]
50	Tyrosine [24]	Amino acid	Aromatic, polar	precursor for thyroid hormones, melanin, and catecholamines [69,70,71,72,73,74]
51	Histidine [24]	Amino acid	Basic (positively charged)	precursor for histamine and involved in immune and antioxidant functions [69,70,71,72,73,74]
52	Ornithine [24]	Amino acid	Basic, non-proteinogenic	key intermediate in the urea cycle and promoter of cell growth and repair [69,70,71,72,73,74]
53	Arginine [24]	Amino acid	Basic (positively charged)	precursor of nitric oxide for vasodilation and immune modulation [69,70,71,72,73,74]
54	Lysine [24]	Amino acid	Basic (positively charged)	essential for collagen formation, calcium absorption, and antiviral defense [69,70,71,72,73,74]
55	Tryptophan [24]	Amino acid	Aromatic, nonpolar	precursor for serotonin, melatonin, and niacin, regulating mood and sleep [69,70,71,72,73,74]
56	Myristic acid [24]	Fatty acid	Saturated fatty acid	antimicrobial, surfactant, membrane-stabilizing, lipid-anchoring, pro-inflammatory modulator, metabolic energy source [75,76,77,78,79]
57	Pentadecanoic acid [24]	Fatty acid	Saturated fatty acid	odd-chain saturated fatty acid, biomarker of dairy fat intake, anti-inflammatory, insulin-sensitizing, cardioprotective [75,76,77,78,79]
58	Palmitic acid [24]	Fatty acid	Saturated fatty acid	major saturated fatty acid in mammals, structural component of membranes, signaling lipid precursor, pro-inflammatory, lipotoxic at high levels [75,76,77,78,79]
59	Palmitoleic acid [24]	Fatty acid	Monounsaturated fatty acid	monounsaturated fatty acid, lipokine activity, anti-inflammatory, improves insulin sensitivity, regulates hepatic lipid metabolism [75,76,77,78,79]
60	Stearic acid [24]	Fatty acid	Saturated fatty acid	long-chain saturated fatty acid, neutral effect on plasma cholesterol, membrane-stabilizing, energy storage, emollient in cosmetics [75,76,77,78,79]
61	Oleic acid [24]	Fatty acid	Monounsaturated fatty acid	monounsaturated ω-9 fatty acid, anti-inflammatory, antioxidant, cardioprotective, improves lipid profile, enhances membrane fluidity [75,76,77,78,79]
62	Linoleic acid [24]	Fatty acid	Polyunsaturated fatty acid	essential ω-6 polyunsaturated fatty acid, precursor of arachidonic acid, maintains skin barrier, regulates inflammation, modulates cell signaling [75,76,77,78,79]
63	Linolenic acid [24]	Fatty acid	Polyunsaturated fatty acid	essential ω-3 polyunsaturated fatty acid, anti-inflammatory, neuroprotective, cardiovascular protective, precursor of EPA and DHA [75,76,77,78,79]

“—” means not found.

## Data Availability

No new data were created or analyzed in this study. Data sharing is not applicable to this article.
